# Extracellular Vesicles in Cardiac Cell Crosstalk: from Intercellular Communication to Clinical Translation

**DOI:** 10.1007/s10557-025-07828-5

**Published:** 2025-12-18

**Authors:** Rahul Sanwlani, Patrizia Camelliti

**Affiliations:** Faculty of Health and Medical Sciences, School of Biosciences, University of Surrey, Guildford, Surrey GU2 7XH United Kingdom

**Keywords:** Cardiovascular diseases, Cardiac extracellular vesicles, Cardiac fibroblast EVs, Cardiomyocytes, Cardiac pathophysiology, Cardiac nonmyocytes

## Abstract

Cardiovascular disease (CVD) remains a leading cause of morbidity and mortality globally, accounting for nearly one-third of deaths worldwide. Intercellular communication between cardiomyocytes and non-cardiomyocytes is fundamental to maintaining cardiac homeostasis and adapting to stress or injury. Among the mediators of this communication, extracellular vesicles (EVs) have emerged as pivotal regulators of cardiac function and remodelling, transporting bioactive molecules that reflect the state and origin of their parent cells.

This review provides a systems-level synthesis of EV-mediated crosstalk in the heart, integrating evidence from cardiomyocyte- and non-cardiomyocyte-derived EVs, including fibroblast, endothelial, vascular smooth muscle, and immune cell sources. We discuss how these vesicles orchestrate signalling networks that influence cardiac remodelling, injury response, and disease progression. Distinct from prior reviews, our article extends beyond mechanistic summaries to explore the translational continuum of cardiac EVs—from their potential as diagnostic and prognostic biomarkers to emerging therapeutic and bioengineering strategies.

Finally, we critically evaluate current technical and regulatory barriers impeding clinical translation, including isolation, characterisation, and validation challenges, and propose a forward-looking roadmap to advance EV-based diagnostics and therapeutics in cardiovascular medicine.

## Cardiovascular Diseases and Extracellular Vesicles

Cardiovascular diseases (CVD) encompass a broad range of disorders affecting the heart and vasculature, including coronary artery disease, heart failure, hypertension, stroke, and peripheral artery disease. As the leading cause of death globally, CVD are responsible for an estimated 17.9 million deaths annually and continue to impose a major public health and economic burden [1, 2]. Despite advances in clinical care and research, prognosis for many patients remains poor, highlighting the urgent need for improved diagnostic tools and more effective therapeutic strategies. In recent years, extracellular vesicles (EVs) have emerged as important players in cardiac pathophysiology, offering exciting potential as novel biomarkers and therapeutic targets for CVD [3, 4].

**Fig. 1:**
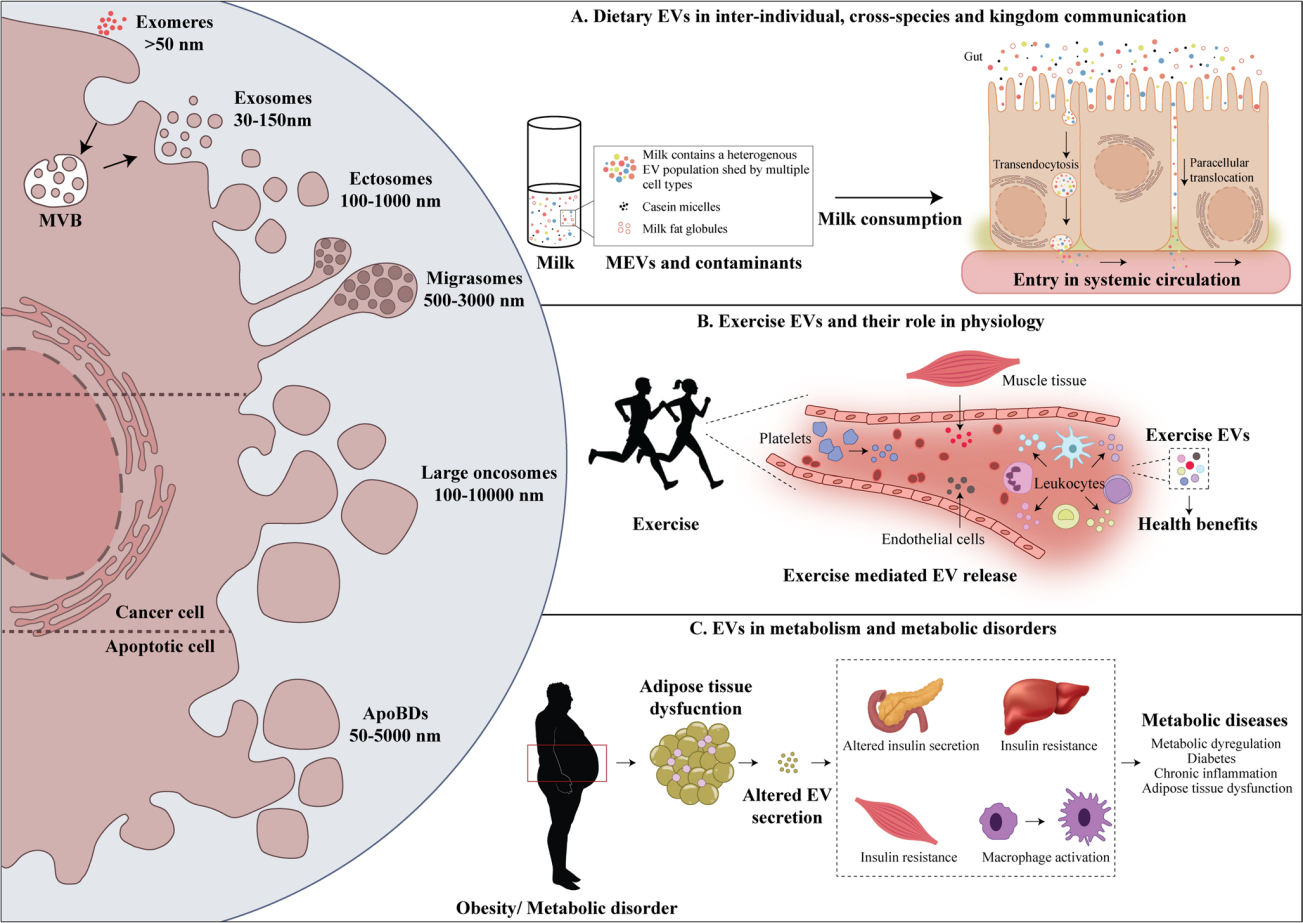
Extracellular vesicles; subtypes and role in pathophysiology. Schematic representation of various EV subtypes classified based on their biogenesis, cell type of origin and size. EVs are understood to be signalling moieties with role in mediating complex pathophysiological events including but not limited to (**A**) Dietary bovine milk EVs regulating systemic phenotypes upon milk consumption, (**B**) Exercise stimulated EVs in plasma imparting health benefits and (**C**) EVs mediating complex metabolic disorders such as diabetes. ApoBDs: Apoptotic bodies

EVs are membrane-bound, non-replicating vesicles secreted into the extracellular space by cells from all life forms. They contain diverse bioactive cargo including proteins, lipids, and nucleic acids that reflect their cell of origin. Eukaryotic EVs are classified into various subtypes based on their biogenesis and origin (Fig. 1) [5–8]. Exosomes (30–150 nm) form through inward budding of endosomal membrane and accumulation of intraluminal vesicles (ILVs) in multivesicular bodies (MVBs). MVBs can either undergo lysosomal degradation or fuse with the plasma membrane to release ILVs as exosomes. Microvesicles (100–1000 nm) or ectosomes originate through direct budding from the plasma membrane via ectocytosis [9, 10]. Apoptotic bodies (1000–5000 nm) are released by apoptotic cells and facilitate clearance of cellular debris through phagocytosis [11]. Additional EV subtypes include migrasomes (500–3000 nm) from migrating cells and large oncosomes (1000–10000 nm) from cancer cells [12, 13].

EVs are increasingly recognised as key mediators of complex signalling events in a variety of pathophysiological processes (Fig. 1) [14–17]. For instance, dietary EVs in bovine milk were observed to regulate cancer progression systemically upon oral administration [18]. The role of EVs stimulated by exercise in deriving health benefits has also been highlighted [19, 20]. Furthermore, cancer cell derived EVs are known to facilitate chemoresistance, metastasis, enhanced cell survival and aid in escaping immune surveillance [21–23]. Contrarily, the pivotal role played by stem cell derived EVs in reducing oxidative stress and apoptosis helps maintain tissue homeostasis and promote overall health [24]. EVs have also been implicated in central nervous system diseases, metabolic disorders like diabetes, and respiratory diseases [17, 25, 26].

Within the cardiovascular system, cells such as cardiomyocytes (CMs), cardiac fibroblasts (CFs), endothelial cells (ECs), vascular smooth muscles cells (VSMCs), resident stem cells, and immune cells actively release EVs. These endogenous cardiac EVs influence a wide range of processes, from maintaining normal cardiac function to contributing to CVD development, progression, or resolution [3, 4]. The significance of cardiac EVs in cardiovascular health is demonstrated by their ability to modulate intercellular communication networks that govern cardiac homeostasis and pathological remodelling.

## Cardiomyocyte and Non-Cardiomyocyte Interactions

The heart is a structurally and functionally complex organ composed of four chambers and a tri-layered wall: the outer epicardium, the thick contractile myocardium, and the inner endocardium [27]. These layers contain a heterogeneous population of cardiac cells, broadly divided into cardiomyocytes (CMs) and non-cardiomyocytes (NCMs). While CMs make up approximately 70–85% of myocardial tissue volume, they represent less than 50% of total cell numbers in the adult heart. The majority of cardiac cells are NCMs, including CFs, ECs, VSMCs, pericytes, epicardial cells, immune cells, and resident progenitor cells [27–33]. NCMs are essential for cardiac function, contributing to extracellular matrix (ECM) regulation, electrical conduction, angiogenesis, immune surveillance, and repair after injury [34, 35].

Effective cardiac function relies on tightly regulated interactions between these different cell types. CMs and NCMs interact through multiple mechanisms, including direct contact via gap junctions, tunnelling nanotubes, and adherens junctions; through mechanical or matrix-mediated signalling; and via the secretion of paracrine and autocrine factors [35–41]. These interactions are especially critical during development, response to stress or injury, and pathological remodelling. For example, epicardial cells provide paracrine cues that influence CM proliferation and maturation during development, and CFs and macrophages modulate CM electrophysiology and contribute to arrhythmogenesis [37, 42–47].

**Fig. 2:**
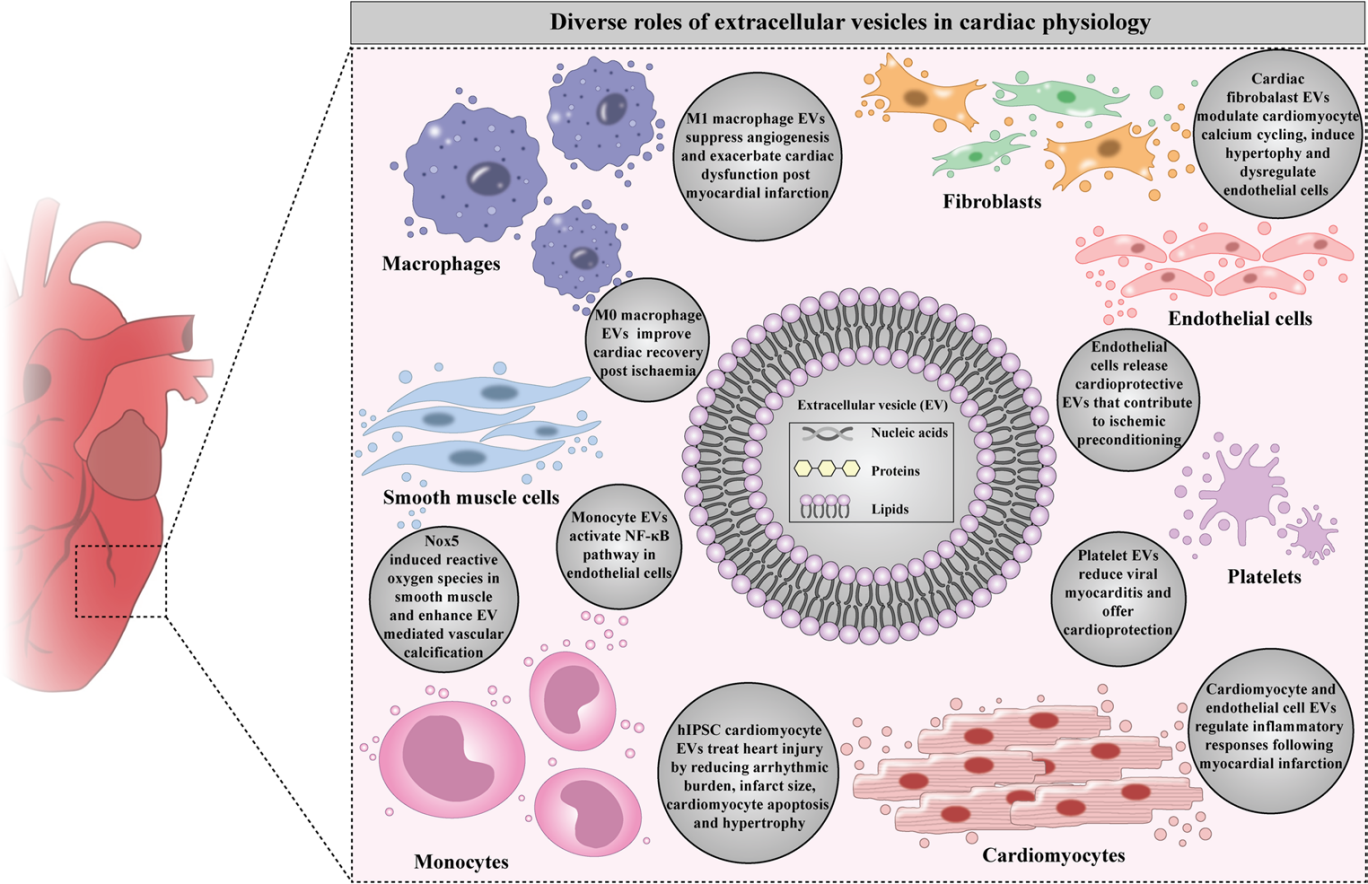
Extracellular vesicles in cardiac pathophysiology. Schematic representation of diverse roles executed by EVs in the heart. Cells of cardiovascular origin including fibroblasts, endothelial cells, macrophages, smooth muscle cells and cardiomyocytes are known to secrete EVs. The EV cargo comprised of bioactives (proteins, lipids and nucleic acids) is dictated by the state of cell type of origin. Depending on the originating cell type and the cargo contained, EVs in the cardiovascular system can have cardioprotective or detrimental effects in recipient cells

While classical paracrine and autocrine signalling mechanisms have long been recognised as key mediators of cardiac cell communication, recent research has highlighted EVs as an additional and potent mode of intercellular signalling within the heart [3, 4, 48]. EVs carry molecular cargo – including proteins, lipids, and nucleic acids – that can influence the behaviour and function of recipient cells in a context-dependent manner. Both CMs and NCMs actively secrete EVs, which operate through autocrine and paracrine routes to regulate processes such as cell survival, inflammation, fibrosis, and tissue repair (Fig. 2; Tables 1 and 2). This EV-mediated communication complements established signalling pathways, adding a new layer of complexity to cardiac cellular crosstalk. Despite growing interest in this field, the specific roles and mechanisms by which EVs mediate interactions between cardiac cell types remain incompletely understood. A deeper mechanistic insight into these processes is essential to fully appreciate how EVs contribute to both cardiac homeostasis and disease progression.

**Table 1:** Cardiomyocyte-derived EVs in cardiovascular pathophysiology

EV donor	EV recipient	Key observations	Cargo responsible	Model	Reference
Rat CM	Mouse cardiac EC	EVs released by CMs isolated from type-2 diabetic rats inhibit EC proliferation, migration and tube-like formation	miR-320	In vitro	[53]
Rat CM	Rat CF	EV mediated CM-CF crosstalk leads to cardiac fibrosis. EV cargo (miR-208a) target CF *Dyrk2* to mediate the effects	miR-208a	In vivo	[54]
Rat neonatal atrial CM	Rat neonatal atrial CF	EVs released by atrial CMs subjected to tachypacing overexpress miR-210-3p which promote atrial CFs proliferation and collagen synthesis by inhibition of GPD1L	miR-210-3p	In vivo	[55]
Rat CM (H9c2)	Rat CM (H9c2)	Post-hypoxia, CMs secrete miR-30a containing EVs which regulate autophagy in CM recipient cells	miR-30a	In vitro	[56]
Neonatal rat CM	Rat EC	Under glucose starvation, CMs secreted EVs which regulated CMEC metabolic activity and increased nutrient uptake	GLUT1 and GLUT4	In vitro	[57]
Rat foetal CM	Mouse EC	EVs released by CMs cultured in ischaemic conditions promote angiogenesis by increasing EC proliferation, sprouting and protecting against oxidative stress	miR-222, miR-143	In vivo	[58]
Neonatal mouse CM	Mouse cardiac EC	EVs containing circHIPK3 released by hypoxia-induced CMs stimulate EC migration, proliferation, and tube formation in vitro, and promote angiogenesis in mouse MI via miR-29a-mediated regulation of VEGFA	CircHIPK3	In vivo	[59]
Mouse CM	Mouse EC	CM HSP20-enriched EVs protect ECs against in vitro hyperglycaemia-triggered cell death and improve cardiac function and angiogenesis in diabetic mice	HSP20	In vivo	[60]
Human CM	Human CMEC	Crosstalk between CMs and CMECs leads to increased survival of CMECs after H/R injury via activation of the eNOS pathway	Linc-ROR	In vivo	[61]
hiPSC-CM	hiPSC-CM and HUVEC	CM-EVs protect hypoxia-stressed CMs and HUVECs in vitro, and in a rat AMI model reduce arrhythmias, improve ejection-fraction, limit infarct size and hypertrophy, and decrease CM apoptosis	Not reported	In vivo	[62]
hiPSC-CM	Human CF	miR-24-3p containing CM EVs demonstrate anti-fibrotic effects	miR-24-3p	In vitro	[63]
Human primary CM	Human CF	CM-EVs revert TGF-β1-activated CFs to less activated state, demonstrating anti-fibrotic activity	Not reported	In vitro	[64]
Mouse CM (HL-1)	Macrophages (RAW 264.7)	CMs exposed to hypoxic conditions release EVs which drive M1 macrophage polarisation	Not reported	In vivo	[65]
Neonatal rat CM	Mouse peritoneal macrophages	Macrophage regulation by CM EVs under basal and pathological (Myocardial ischaemia) conditions leading to cardiac homeostasis and pro-inflammatory effects	Not reported	In vitro	[63, 66]
Rat CM (H9c2)	Macrophages (RAW264.7)	EVs from hypoxic CMs promote anti-inflammatory polarisation (M2) in recipient macrophages	Not reported	In vitro	[63, 67]

**Table 2:** Non-cardiomyocyte-derived EVs in cardiovascular pathophysiology

EV donor	EV recipient	Key observation	Cargo responsible	Model	Reference
Cardiac fibroblast-derived EVs in cardiovascular pathophysiology					
Rat and mouse neonatal CF	Rat neonatal CM	EVs released by AngII-activated CFs induce CM hypertrophy via upregulation of RAS and activation of MAPKs and Akt.	Not reported	In vivo	[74]
Rat neonatal CF	Rat neonatal CM	CM hypertrophy caused by AngII-activated CF-EVs targeting PDLIM5 in CMs.	miR-27a*	In vivo	[75]
Rat neonatal CF	Rat neonatal CM	CM hypertrophy mediated by miRNA21-3p targeting SORBS2 and PDLIM5 in CMs.	miR-21-3p	In vivo	[76]
Rat CF	Rat CM (H9c2)	EVs released by TNF-α-treated CFs downregulate the Nrf2/ARE signalling pathway and promote hypertrophy in CMs.	miR-27a, miA-28-3p, and miR-34a	In vivo	[77]
Rat neonatal CF	Mouse EC	TGF-β1 treated CF-EVs lead to EC dysregulation - reduced migration and proliferation and increased apoptosis.	miRNA-200a-3p	In vitro	[78]
Rat neonatal CF	Rat neonatal CF	Senescent CF-EVs reduce live cell counts and proliferation in recipient naïve CFs, and induced CFs autophagy via the suppression of RHEBL1 protein.	Not reported	In vitro	[79, 80]
Rat neonatal atrial CF	Rat neonatal atrial CM	EVs released by AngII-activated CFs downregulate Ca_v_1.2 expression in CMs.	Not reported	In vitro	[81]
Human primary CF	hiPSC-CM	EVs from end-stage heart failure CFs affect CM calcium transients.	Not reported	In vitro	[82]
Rat atrial CF	Rat neonatal atrial CM	EVs released by AngII-activated CFs promote an atrial fibrillation phenotype by inhibiting CACNA1c expression in CMs.	miR-224-5p	In vivo	[83]
Rat neonatal CF	Rat neonatal CF	Hypoxia and isoflurane induced CF-EVs increase migration of recipient CFs.	Not reported	In vitro	[84]
Mouse neonatal CF	Rat CM (H9c2)	Under stress conditions induced with hypoxia and reoxygenation, CFs secrete EVs enriched with miR-133a. These EVs target ELAVL1 in recipient CMs and prevent pyroptosis.	miR-133a	In vitro	[85]
Rat neonatal CF	Rat CM (H9c2)	Hypoxia/reoxygenation post-conditioning led to release of miR-423-3p enriched EVs by CFs which protected CMs from hypoxia/reoxygenation injury.	miR-423-3p	In vivo	[86]
Endothelial cell-derived EVs in cardiovascular pathophysiology					
Mouse EC	Mouse neutrophils	TNF-α activated ECs produce EVs enriched for VCAM-1 and miR-126. These EVs mobilise splenic neutrophil reserves to peripheral blood, which correlates with myocardial injury in AMI.	miR-126	In vivo	[99]
Mouse CMEC	Mouse neonatal CF	Under hyperglycaemia, CMECs release TGF-β1 mRNA enriched EVs which mediate CFs activation.	TGF-β1 mRNA	In vitro	[100]
Mouse CMEC	Mouse neonatal CM	Mst1-enriched EVs released from CMECs inhibit autophagy, enhance apoptosis and suppress glucose metabolism in recipient CMs cultured in high glucose conditions.	Mst1	In vitro	[101]
HUVEC	Rat neonatal CM	Under peripartum cardiomyopathy induced conditions, ECs release miR-146a-enriched EVs which attenuate angiogenesis and suppress CMs metabolic activity.	miR-146a	In vitro	[102]
Human microvascular EC (HMEC-1)	Human microvascular EC (HMEC-1)	ECs secrete EVs containing miR-214, which suppress senescence and stimulates an angiogenic program in recipient ECs.	miR-214	In vivo	[104]
Human EC	Human EC	Atherosclerotic conditions promote packaging of miR-92a-3p in EC-EVs, which regulates angiogenesis in recipient ECs by a THBS1-dependent mechanism.	miR-92a-3p	In vitro	[105]
HUVEC, Mouse EC	Human aortic VSMC	KLF2-transduced or shear-stress-stimulated HUVECs release EVs enriched in miR-143/145 which regulate VSMC phenotype in vitro and protect against atherosclerotic lesion in vivo.	miR-143/miR-145	In vivo	[106]
HUVEC	Rat CM	EC-EVs reduce CM cell death after hypoxia/reperfusion injury via activation of the ERK1/2 MAPK signalling pathway.	Not reported	In vitro	[107]
Human EPC	Human CF	EPC-EV miRs promote phenotypic changes of CFs to ECs, enhancing angiogenesis in vitro and in vivo, and improving post-MI repair in rats.	miR-1246, miR-1290	In vivo	[108]
HUVEC	Human monocytes, (THP-1)	EC-EVs modulate macrophage polarization toward pro- or anti-inflammatory phenotypes, depending on atherosclerotic context.	miR-155	In vivo	[109]
HUVEC, Human coronary artery EC	Human monocytes (THP-1), Human primary monocytes	Quiescent EC-EVs regulate monocyte signalling mechanisms by supressing NF-κB pathway and exert anti-inflammatory effects.	miR-10a	In vivo	[110]
Rat aortic EC	Rat monocytes	HSP70 dependent activation of monocytes by EC-EVs.	HSP70	In vitro	[111]
Vascular smooth muscle cell-derived EVs in cardiovascular pathophysiology					
Human aortic VSMC	Calcifying effect	Role in calcification.	Not reported	In vivo	[120]
Rat primary VSMC	Rat primary VSMC	Calcifying VSMC release EVs that accelerate calcification of recipient normal VSMC.	Not reported	In vitro	[121]
Human aortic VSMC	HUVEC	VSMC-EVs inhibit autophagy in recipient ECs.	miR-221/222	In vitro	[126]
Human aortic VSMC	Primary ECs	VSMC-ECs increased EC migration	Not reported	In vitro	[124]

This review focuses on the role of cardiac cell-derived EVs in intercellular signalling and cardiac pathophysiology. In the following sections, we examine EVs released by CMs and NCM populations (including CFs, ECs, VSMC cells, immune cells, and platelets), highlighting their contributions to cardiovascular health and disease. While several previous reviews have discussed EVs in cardiovascular contexts, most have focused on individual cardiac cell types or general EV biology. In contrast, this review adopts an integrative, systems-level approach that collectively examines CM- and NCM-derived EVs within the broader framework of multicellular cardiac crosstalk. Furthermore, by extending beyond descriptive mechanisms to address translational barriers, bioengineering innovations, and regulatory considerations, we aim to provide a forward-looking perspective that bridges fundamental EV biology with clinical translation in cardiovascular medicine. Specifically, we discuss how cardiac EVs are being explored as diagnostic biomarkers and therapeutic vectors, and we highlight key challenges and innovation pathways necessary to advance their application in cardiovascular disease.

## Cardiomyocyte-Derived EVs and their Role in Cardiac Pathophysiology

Although CMs are the most metabolically active cell type in the heart, driving its contractile function, they were long considered relatively less secretory than other cardiac cells, such as CFs [3, 49]. Over the past two decades, however, increasing evidence has revealed the significance of CM-derived EVs, particularly their cargo composition and functional roles in cardiac pathophysiology [3, 50, 51].

CM-derived EVs carry a diverse repertoire of biomolecules, including proteins, miRNAs, and metabolic regulators, with their composition and secretion dynamics influenced by the microenvironment and pathological state of the cells [3, 50, 51]. Notably, they are enriched in heat shock proteins (HSP20, HSP60, and HSP70) which contribute to CM survival and stress adaptation. Some CM-EVs also contain inflammatory mediators such as interleukin-6 (IL-6) and tumor necrosis factor-alpha (TNF-α), suggesting a role in modulating inflammatory responses and cardiac remodelling. Depending on the physiological or pathological context, CM-EVs may carry molecular regulators that influence diverse cardiac and vascular cell types [3, 52].

CM-EVs are actively taken up by various cardiac cell populations, including ECs and CFs, modulating their functions (Table 1) [53–67]. Several studies implicate CM-EVs in disease progression. In a rat model of diabetic cardiomyopathy, CM-EVs enriched with miR-320 suppressed HSP20 in ECs, impairing migration and proliferation, exerting anti-angiogenic effects [53]. Similarly, hypoxic or post-AMI CMs secrete EVs enriched in miR-208a, which, upon uptake by CFs, promote myocardial fibrosis [54]. Furthermore, atrial CMs subjected to tachypacing have been shown to release miR-210-3p-enriched EVs, which promote atrial CF proliferation and collagen synthesis via GPD1L signalling [55]. CM-EVs can also act in an autocrine manner; for example, under hypoxia, they carry high levels of miR-30a, which promotes autophagy in recipient CMs by regulating Beclin-1 and ATG12 [56].

Despite these pathological roles, CM-EVs can also mediate cardioprotection under specific conditions. Under glucose starvation, CM-EVs are enriched with glucose transporters (GLUT1, GLUT4) and glycolytic enzymes, facilitating CM–EC metabolic crosstalk that increases glucose transfer to CMs during stress [57]. Post-ischemic CM-EVs have been shown to promote angiogenesis by stimulating EC proliferation and migration via miR-222/miR-143 or circHIPK3-enriched cargo [58, 59]. CM-EVs have also been reported to increase EC survival in hypoxia-reoxygenation and hyperglycaemia-induced stress, via the activation of eNOS signalling or HSP20-mediated mechanisms respectively [60, 61]. CM-EVs enriched in cardiac-specific miRNAs, including miR-1 and miR-133a, exert cardioprotective effects on CMs and ECs exposed to hypoxia in vitro, while improving cardiac function and reducing infarct size and hypertrophy in vivo in a rat AMI model [62]. Similarly, miR-24-3p–enriched CM-EVs inhibit CF activation and ECM secretion [63]. More recently, in vitro and in vivo studies have shown that CM-EVs can reduce CFs activation, ECM deposition, and overall fibrosis while promoting angiogenesis [64].

Beyond direct effects on cardiac cells, CM-EVs influence immune regulation. Under homeostatic conditions, they help maintain cardiac immune balance via macrophage interactions. In pathological states such as myocardial infarction and ischemia, CM-macrophage crosstalk shifts macrophages toward a pro-inflammatory phenotype, contributing to maladaptive remodelling and disease progression [65, 66]. Conversely, in other models, hypoxic CM-EVs promoted M2 macrophage polarisation, mitigating CM injury through anti-inflammatory mechanisms [67].

Collectively, these findings illustrate the dual nature of CM-derived EVs as mediators of pathological remodelling on one hand and facilitators of cardioprotection and repair on the other. Their functional heterogeneity is dictated by the cellular environment, disease state, and EV cargo composition. A deeper understanding of these mechanisms may enable the development of CM-EV–based therapeutics and diagnostics for CVD.

## Non-Cardiomyocyte-Derived EVs in Cardiac Pathophysiology

The abundance and composition of cardiac cell types vary dynamically between physiological and pathological states, as well as across developmental stages from embryogenesis to adulthood. For instance, while the number of CMs is largely established during the perinatal period, the CF population continues to expand postnatally, eventually comprising approximately 15–25% of total cardiac cells in the mature heart. Similarly, immune cells (3–16%) and epicardial cells (0.5–5.5%) increase in number during development, whereas the proportion of ECs (10–20%) decreases [27–33, 68]. In the adult human heart, NCMs account for approximately 65% of all cardiac cells, underscoring their essential roles in regulating CM function and, consequently, cardiac pathophysiology [27, 29, 31, 33]. Under physiological conditions, NCM-derived EVs contribute to maintaining cardiac homeostasis. However, depending on their cell of origin and the prevailing disease state, NCM-EVs can also facilitate maladaptive processes, thereby contributing to the onset and progression of CVDs [3, 4, 48, 52].

## Cardiac Fibroblast-Derived EVs

CFs represent a major NCM population in the heart, where they are central to the maintenance of cardiac homeostasis and to structural and functional remodelling in response to stress conditions such as ischemia. They influence CM phenotype and function, thereby modulating cardiac performance under stress [69, 70]. Cardiac remodelling triggered by pathological stress is characterised by fibrosis and CM hypertrophy, changes that can ultimately lead to heart failure. During these events, CFs proliferate and influence CMs function via both autocrine and paracrine mechanisms, driven by excessive secretion of ECM proteins and proinflammatory cytokines [37, 71–73].

More recently, CF-derived EVs (CF-EVs), and in particular their miRNA cargo, have emerged as mediators of CM hypertrophy [74–77] (Table 2). CF-EVs are readily internalised by CMs in vitro, highlighting their role in intracardiac communication. For example, angiotensin II-stimulated CFs increase EV secretion; once taken up by CMs, these EVs elevate angiotensin II levels, upregulate its receptors AT1R and AT2R, and activate the RAS, MAPKs, and Akt pathways, ultimately driving CM hypertrophy [74]. Stress conditions can also alter the selective miRNA packaging into CF-EVs. For instance, miR-21-3p and miR-27a* are enriched in CF-EVs during cardiac stress and promote hypertrophy in recipient CMs [75, 76]. Similarly, miR-27a, miR-28-3p, and miR-34a are upregulated in TNF-α-stimulated CF-EVs and contribute to CM hypertrophy via the Nrf2/ARE signalling pathway [75, 77].

CF-EVs can also affect other cardiac cell types. For example, TGF-β1–treated CFs secrete EVs enriched in miR-200a-3p, which impair EC function by increasing apoptosis and reducing proliferation and migration [78]. Autocrine effects have also been described: EVs from doxorubicin-induced senescent CFs exhibit reduced protein cargo and, when transferred to naïve CFs, decrease viability, inhibit proliferation, and trigger autophagy via suppression of RHEBL1 [79, 80].

Beyond structural remodelling, CF-EVs have been proposed as potential modulators of CM electrophysiology, possibly influencing calcium handling in recipient CMs, although the underlying mechanisms and physiological relevance remain to be fully elucidated [81–83].

Although most studies highlight detrimental roles for CF-EVs in cardiac pathology, emerging evidence suggests that under specific physiological or stress-adaptive conditions, they can also exert cardioprotective effects (Table 2). For instance, in vitro preconditioning of CFs with hypoxia or isoflurane alters EV cargo composition, yielding CF-EVs enriched in cardioprotective proteins and miRNAs that enhance migration of non-conditioned CFs [84]. Similarly, CFs subjected to hypoxia/reoxygenation release EVs enriched with miR-133a, which protect CMs from ischemia/reperfusion injury by targeting ELAVL1 and suppressing pyroptosis [85]. Ischaemia postconditioning has also been shown to stimulate the release of miR-423-3p–enriched CF-EVs, which improve CM viability and reduce apoptosis [86]. These findings indicate that, under certain stress-adaptive conditions, CF-EVs may function as endogenous mediators of myocardial protection.

Collectively, current evidence supports an extensive and multifaceted role for CF-EVs in autocrine and paracrine signalling, both in health and disease. By modulating CM phenotype, influencing other NCM populations, and integrating into broader cardiac communication networks, CF-EVs expand the functional repertoire of CFs well beyond their traditional roles in ECM production and fibrosis.

## Endothelial Cell-Derived EVs

The vascular endothelium is essential for cardiovascular homeostasis, maintaining the vascular barrier, regulating blood vessel tone, and enabling immune cell trafficking through transient openings. In addition to soluble mediators, ECs release EVs (EC-EVs) that influence cardiovascular health and disease [87–89]. Under physiological conditions, quiescent ECs release EVs at low levels. However, cardiovascular risk factors such as diabetes, hypertension, cigarette smoke, environmental toxins, and ionizing radiation can disrupt this balance, leading to chronic endothelial injury. This injury is characterized by EC activation, apoptosis, vascular dysfunction, and atherosclerosis, which are key drivers of CVDs [87, 90, 91].

Elevated EC-EV levels are strongly associated with vascular dysfunction and increase in response to myocardial injury. In vitro, EC activation by angiotensin II, TNF-α, or plasminogen markedly increases EV secretion [92–95]. In vivo, plasma EC-EV levels are low in healthy individuals but elevated in acute and chronic CVDs associated with endothelial dysfunction [87]. Clinical studies highlight their potential as disease biomarkers: in a cohort of 844 patients with CVD complications, higher EC-EV levels correlated with cardiometabolic risk [96]; elevated levels have also been reported in obesity, end-stage renal failure and following hypoxic exposure in human blood samples [90, 97, 98].

Whether EC-EV release is a cause or a consequence of disease remains unclear. Their prognostic value likely depends on cargo composition and functional effects [87]. Both detrimental and protective roles have been described (Table 2). Pathological effects include VCAM-1⁺ EC-EVs exacerbating myocardial infarction by accelerating neutrophil mobilization and enlarging infarct size [99]; TGF-β1 mRNA–loaded EVs from CMECs activating CFs under hyperglycaemia [100]; and Mst1-enriched CMEC-EVs regulating CM autophagy, glucose metabolism, and apoptosis in diabetic cardiomyopathy models [101]. In peripartum cardiomyopathy, miR-146a–enriched EC-EVs impaired angiogenesis and reduced CM metabolic activity [102].

Conversely, EC-EVs can promote vascular repair and cardioprotection. Exercise-derived EVs represent another promising source of cardioprotective signals. Long-term exercise-derived circulating exosomes have been shown to convey cardioprotective signals, with exosomal miR-342-5p identified as a novel cardioprotective exerkine against myocardial ischemia/reperfusion injury in rats [103]. This highlights the potential therapeutic value of exercise-induced EV modulation as a non-pharmacological approach to cardiovascular protection. Proangiogenic examples include miR-214–containing EC-EVs that suppress senescence and stimulate EC migration and angiogenesis [104], and miR-92a-3p–enriched EC-EVs in coronary artery disease patients that enhance angiogenesis in recipient ECs [105]. Under atherosclerotic conditions, miR-143/145–rich EC-EVs modulate VSMC phenotype, exerting atheroprotective effects [106]. EC-EVs can confer resistance to hypoxia/reoxygenation injury in CMs via the activation of the ERK1/2 MAPK signalling pathway [107]. Endothelial progenitor cell (EPC)-derived EVs also show therapeutic promise, with miR-1246 and miR-1290 cargo promoting phenotypic changes of CFs to ECs, enhancing angiogenesis and ameliorating cardiac injury and fibrosis after MI [108].

EC-EVs also modulate immune responses in CVDs (Table 2). Oxidized LDL–stimulated EC-EVs enriched in miR-155 drive macrophage polarization toward a proinflammatory M1 phenotype, whereas KLF2-overexpressing EC-EVs shift polarization toward the anti-inflammatory M2 phenotype [109]. Quiescent EC-EVs can transfer miR-10a to monocytes, suppressing NF-κB signalling and exerting anti-inflammatory effects [110]. In contrast, HSP70-containing EC-EVs activate monocytes and promote their adhesion to ECs [111].

In summary, EC-EVs exert highly context-dependent effects, ranging from vascular injury and inflammation to angiogenesis and myocardial protection. Their dual roles underscore the need for deeper mechanistic studies to harness EC-EVs as both biomarkers and therapeutic agents in CVDs.

## Vascular Smooth Muscle Cell-Derived EVs

VSMCs are the most abundant cell type in the vasculature and display remarkable phenotypic plasticity [112, 113]. In their mature state, VSMCs are typically quiescent, with low secretory activity, and primarily maintain vessel wall integrity, produce ECM components, and regulate arterial tone, thereby supporting both the structural and conductive roles of the vasculature [112, 114].

Under pathological conditions such as oxidative stress or mechanical injury, VSMCs undergo phenotypic switching from a contractile to a synthetic, non-contractile state characterised by enhanced secretory activity [112–115]. This transition is further promoted by oxidised LDLs (oxLDLs), pro-inflammatory cytokines, and growth factors released by neighbouring cells via paracrine signalling. Activated VSMCs show increased proliferation and migration, contribute to ECM remodelling, and may adopt osteogenic, chondrogenic, or inflammatory phenotypes. In this state, VSMCs also release EVs (VSMC-EVs), which have been implicated in vascular pathologies including atherosclerosis, vascular calcification, and coagulation [114, 116, 117] (Table 2).

Several studies have linked VSMC-EVs to phenotype switching and vascular calcification. Synthetic-phenotype VSMC-EVs are enriched in annexins, tissue non-specific alkaline phosphatase, and phosphatidylserine, and share similarities with osteoblast-derived EVs, including calcium-binding properties that act as nucleation sites for calcium phosphate deposition [114, 116–118]. Under physiological conditions, VSMC-EVs contain calcification inhibitors such as matrix Gla protein and fetuin-A. However, pathological stress, including mineral imbalance and inflammatory cues, depletes these inhibitors and promotes calcification. For example, elevated extracellular calcium disrupts VSMC homeostasis, leading to reduced calcification inhibitors and increased calcium loading in EVs, which accelerates calcification [114, 119–121].

VSMC-EVs also contribute to vascular inflammation and atherosclerosis. Bidirectional EV-mediated cross-talk between VSMCs and neighbouring cells is well documented in the vascular/cardiovascular context: EC-derived EVs alter VSMC phenotype and inflammatory state (including HMGB proteins and miRNAs), and macrophage/foam-cell EVs can increase VSMC migration and adhesion — processes central to atherogenesis and plaque progression [120, 122, 123]. VSMC-EVs themselves can directly influence ECs; for example, PDGF-stimulated VSMCs secrete EVs with altered miRNA cargo (reduced miR-1246, miR-182, miR-486) that enhance EC migration [124], while PCSK9-overexpressing VSMCs release EVs that induce endothelial activation and inflammatory responses [125]. In another study, VSMC-EVs containing miR-221/222 altered autophagy signalling in ECs, making them more susceptible to atherogenic stimuli [126]. Notably, while several studies confirm VSMC-EVs are present in the cardiac/vascular milieu and that they act on ECs and immune cells, direct evidence that coronary VSMC-derived EVs deliver functional cargo to CMs in situ remains limited, representing an important gap for future coronary-specific EV tracing and functional studies.

Collectively, these findings highlight the functional versatility of VSMC-EVs as mediators of vascular remodelling, calcification, and intercellular communication in disease. A deeper mechanistic understanding of their biogenesis, cargo sorting, and target cell specificity could uncover novel diagnostic biomarkers and therapeutic targets for vascular and cardiac pathologies.

## Immune Cell-Derived EVs in Cardiovascular Pathophysiology

Previous sections have examined the roles of EVs from endogenous cardiac cells, particularly CMs and NCMs in regulating cardiac function and contributing to CVDs. This section shifts focus to immune cell–derived EVs, specifically those released by macrophages, neutrophils, and platelets. Cardiac-resident macrophages are present under physiological conditions, while monocyte-derived macrophages are rapidly recruited in response to inflammation or injury [127–129]. Platelets and neutrophils, although not native to the myocardium, are likewise swiftly mobilised to sites of cardiac injury or inflammation [99, 130]. EVs from these immune cells have emerged as important modulators of cardiovascular responses, influencing inflammation, fibrosis, and remodelling [127–132].

## Cardiac Macrophage EVs

Cardiac macrophages, though representing only a small fraction of the stromal population, play a crucial role in both injury and repair in CVDs [27, 46, 127–129]. Two main subpopulations have been identified: CCR2⁻ macrophages, derived from embryonic lineages, and CCR2⁺ macrophages, derived from definitive haematopoiesis. Their origin determines their function: embryonic macrophages regulate development, maintenance, repair, and adaptive remodelling, whereas CCR2⁺ macrophages respond to injury, drive inflammation and fibrosis, and are implicated in arrhythmias and aging-related pathologies.

A dynamic interplay exists between resident and circulating monocyte-derived macrophages. Following cardiac injury or during CVD, cardiac macrophages proliferate and secrete chemotactic factors that recruit monocytes. In the early post-MI phase, monocytes differentiate into pro-inflammatory M1 macrophages, which clear apoptotic CMs via phagocytosis, MHC-II antigen presentation, and ROS production. In later stages, a phenotypic switch occurs toward reparative M2 macrophages, which support ECM deposition, angiogenesis, proliferation, and tissue remodelling [127, 128, 133–135]. Recent studies highlight macrophage-derived EVs as central mediators of these processes (Table 3).

**Table 3:** Immune cell-derived EVs in cardiovascular pathophysiology

EV donor	EV recipient	Key observation	Crago responsible	Model	Reference
Mouse macrophages (bone marrow derived macrophages: BMDM)	Mouse neonatal CM	M1-EVs mediate CM pyroptosis	miR-29a	In vitro	[136]
Mouse BMDM	Mouse CF	M1-EVs suppress CF proliferation and promote CF inflammation	miR-155	In vivo	[137]
Mouse macrophages (RAW 264.7)	Mouse neonatal CM	M1-EVs suppress CM proliferation via the IL-6R/JAK/STAT pathway	miR-155	In vivo	[138]
Human (THP-1) and mouse (RAW264.7, BMDM) macrophages	Human coronary artery EC	M1-EVs decrease EC proliferation and angiogenesis in vitro, and exacerbate cardiac dysfunction in a mouse MI model	miR-155	In vivo	[139]
Human macrophages (differentiated from U937)	HUVEC	M1-EVs promote EC injury	miR-4532	In vitro	[140]
Mouse M1 BMDM	Mouse CMEC	M1-EVs impair angiogenesis and myocardial repair following MI	lncRNA MALAT1	In vivo	[141]
Rat BMDM	Rat CM (H9C2)	M2-EVs attenuate hypoxia/reoxygenation-induced CM injury in vitro and suppress oxidative stress, inflammation and pyroptosis in vivo	Not reported	In vivo	[142]
Rat macrophages (peritoneal)	Rat neonatal CM	M2-EVs enhance viability of CMs and reduce infarct size in a model of ischemia/reperfusion injury	miR-148a	In vivo	[143]
Mouse BMDM	Mouse CM (HL-1)	M2-EVs prevent CM pyroptosis post-MI via downregulation of ELAVL1 gene	miR-378a-3p	In vivo	[144]
Human macrophages (differentiated from peripheral blood monocytes)	Human CM (AC16)	M2-EVs reduce CM apoptosis and alleviate AMI injury via SOX6 downregulation	miR-1271-5p	In vivo	[145]
Human macrophages (differentiated from peripheral blood monocytes)	Human CM (AC16)	M2-EVs carrying miR-145-5p inhibit CM pyroptosis via TLR4 downregulation	miR-145-5p	In vivo	[146]
Human macrophages (THP-1)	Human coronary artery EC, human artery EC, HUVEC	M2-EVs promote angiogenesis via thrombospondin-1 (THBS1) mRNA suppression	miR-132-3p	In vivo	[147]
Human macrophages (THP-1)	HUVEC	M2-EVs increase ECs proliferation and reduce apoptosis/inflammation via suppression on the target gene Grb10	miR-221-3p	In vitro	[148]
Mouse BMDM	Mouse Primary CF	M2-EVs exacerbate myocardial fibrosis and maladaptive cardiac remodelling	CircUbe3a	In vivo	[149]
Human neutrophils	Not reported	Neutrophil-EVs aggravate sepsis induced cardiomyopathy	miR-150-5p	In vivo	[153]
Human neutrophils	HUVEC, Human dermal microvascular EC	Neutrophil-EVs induce pro-inflammatory responses in ECs and cause vascular damage	miR-223‐3p, miR-142-3p and miR-451	Ex vivo	[154, 155]
Human neutrophils	Human arterial EC	Neutrophil-EVs drive EC inflammation and atherosclerosis via NF-κB activation	miR-155	In vitro	[156]
Human platelets	Rabbit aorta EC	Under sepsis-like stimuli platelets-EVs induce EC apoptosis	Not reported	In vitro	[157]
Rat platelets	Rat aortic EC	Platelets-EVs induce EC apoptosis via suppression of BCL2L1	miR-142-3p	In vitro	[158]
Human platelets	HUVEC	Platelets-EVs induce EC apoptosis via reduction of insulin-like growth factor-1 receptor	miR-223	In vitro	[159]
Rat platelets	Rat aortic EC	Platelets-EVs promote EC proliferation	miR-142-3p	In vitro	[160]
Human platelets	Human macrophage	Platelet-EVs prevent macrophage migration to cardiac tissue post AMI	miR-4306	In vitro	[164]
Human platelets	Human CMEC, Rat CM (H9C2), Human CM (AC16)	Platelet-EVs exhibit cardioprotective effects- improve cardiac function, reduce scar formation and enhance angiogenesis post MI	Not reported	In vivo	[165]
Human platelets	Not reported	Platelet-EVs from healthy donors reduce myocarditis in mice and improve cardiac function	Not reported	In vivo	[166]

Macrophage-derived EVs exert either detrimental or reparative effects depending on polarization state. M1-derived EVs (M1-EVs) are generally pathogenic and exacerbate cardiac injury. For instance, miR-29a–enriched M1-EVs induce CM pyroptosis [136], while miR-155–containing M1-EVs activate pro-inflammatory pathways in CFs and inhibit CM proliferation [137, 138]. M1-EVs also impair endothelial function and hinder repair post-injury. Uptake of miR-155–rich M1-EVs by ECs reduces viability, proliferation, and tube formation in vitro, and intramyocardial injection of M1-EVs suppresses angiogenesis and worsens injury in a mouse MI model [139]. Similarly, M1-EVs carrying miR-4532 disrupt EC function by targeting specificity protein 1 (SP1) and activating downstream NF-κB p65 signalling [140]. Moreover, M1-EVs enriched in the long non-coding RNA MALAT1 impair angiogenesis and myocardial recovery both in vitro and in vivo, acting via the MALAT1/miR-25-3p/CDC42 axis and MEK/ERK pathway activation [141].

By contrast, M2 macrophage-derived EVs (M2-EVs) predominantly exert protective effects and support myocardial repair. M2-EVs protect CMs from ischemia-reperfusion and MI injury by reducing oxidative stress, inflammation, apoptosis, and pyroptosis [142–146]. These effects are largely mediated through delivery of protective miRNAs (e.g., miR-148a, miR-378a-3p, miR-1271-5p, miR-145-5p), which target pathways such as TXNIP/NLRP3, ELAVL1, SOX6, and TLR4 to enhance cell viability and promote cardiac repair. M2-EVs also exert therapeutic effects on ECs. Upon uptake by ECs, miR-132-3p–rich M2-EVs increase viability, proliferation, migration, and tube formation in vitro while enhancing angiogenesis and cardiac function in vivo following MI [147]. Similarly, M2-EVs carrying miR-221-3p promote EC proliferation and inhibit apoptosis and inflammation through suppression of the target gene growth factor receptor binding protein-10 (Grb10) [148]. In contrast, M2-EVs carrying the circular RNA CircUbe3a have been reported to promote proliferation, migration, and activation of CFs by targeting the miR-138-5p/RhoC axis, ultimately leading to myocardial fibrosis and maladaptive remodelling [149].

Together, these findings highlight cardiac macrophage-derived EVs as pivotal modulators of cardiac pathophysiology. Depending on polarization state, they can either exacerbate injury or promote repair, making them attractive targets for therapeutic intervention in CVD.

## Neutrophil and Platelet EVs

Neutrophils and platelets, while not cardiac-resident under physiological conditions, are rapidly recruited to sites of cardiac injury or inflammation in response to pathological stimuli [99, 130]. These cells infiltrate the myocardium during acute and chronic CVDs, including MI, heart failure and ischemia reperfusion injury, where they contribute to immune regulation, tissue remodelling, and disease progression [150–152]. EVs from neutrophils and platelets have emerged as dynamic regulators of cardiac pathophysiology, serving as critical mediators between innate immune activation and cardiovascular outcomes [114]. These EVs modulate endothelial function, immune cell recruitment, coagulation pathways, and fibroblast activity, exerting either detrimental or protective effects depending on the pathological context (Table 3).

Neutrophil-derived EVs (neutrophil-EVs) are primarily associated with inflammatory and detrimental effects in CVDs. In sepsis-induced cardiomyopathy, neutrophil-EVs function as disease mediators through delivery of miR-150-5p [153]. Similarly, neutrophil-EVs containing miR-223‐3p, miR-142-3p, or miR-451 induce endothelial inflammatory cascades and vascular damage upon internalization by ECs, disrupting critical signalling pathways including ERK1/2 and eNOS-mediated mechanisms [154, 155]. Furthermore, in response to high-fat diet, neutrophils-EVs contribute to vascular inflammation and atherogenesis through delivery of miR-155 to ECs, which enhances NF-κB activation [156].

Platelets-derived EVs (platelets-EVs) exhibit complex effects on cardiovascular pathophysiology, with their diverse cargo composition contributing to varied biological outcomes. Under pathological conditions, platelet EVs can exert detrimental effects on endothelial function. In response to sepsis-like stimuli, platelet-EVs induce EC caspase-3 activation and apoptosis through generation of superoxide, NO and peroxynitrite [157]. These effects could be mediated through pro-apoptotic cargo, particularly miR-142-3p and miR-223, which have been shown to promote apoptosis upon uptake by ECs via suppression of the target gene BCL2L1 and reduction of insulin-like growth factor-1 receptor, respectively [158, 159]. In hypertensive rats, elevated levels of miR-142-3p in platelet-EVs are associated with enhanced EC proliferation via suppression of BCL2-associated transcription factor (BCLAF1), which could promote endothelial dysfunction [160].

Conversely, platelet-EVs demonstrate protective cardiovascular effects under certain conditions. Platelet-EVs are enriched in proteasome/ubiquitin system components that confer anti-inflammatory properties [161–163]. Following MI, platelets-EVs carrying miR-4306 prevent macrophage migration to cardiac tissue through VEGFA/ERK1/2/NF-κB signalling pathways [164]. The cardioprotective potential of platelet-EVs has been further demonstrated using clinical-grade human platelet concentrates, which improve cardiac function, reduce scar formation, and enhance angiogenesis in mouse ischemia-reperfusion models while promoting CM activity and metabolism, reducing apoptosis, and enhancing EC tube formation in vitro [165]. Additionally, platelet-EVs from healthy donors alleviate myocarditis and improve cardiac function through enrichment of anti-inflammatory cargo components [166].

Together, these findings establish neutrophil- and platelet-EVs as influential modulators of cardiac pathophysiology. Their capacity to regulate endothelial function, modulate inflammatory responses, and influence tissue repair positions them as both pathological mediators and therapeutic targets, with their specific effects determined by disease context and cargo composition.

## Clinical Relevance of Cardiac EVs and Current Limitations

**Fig. 3:**
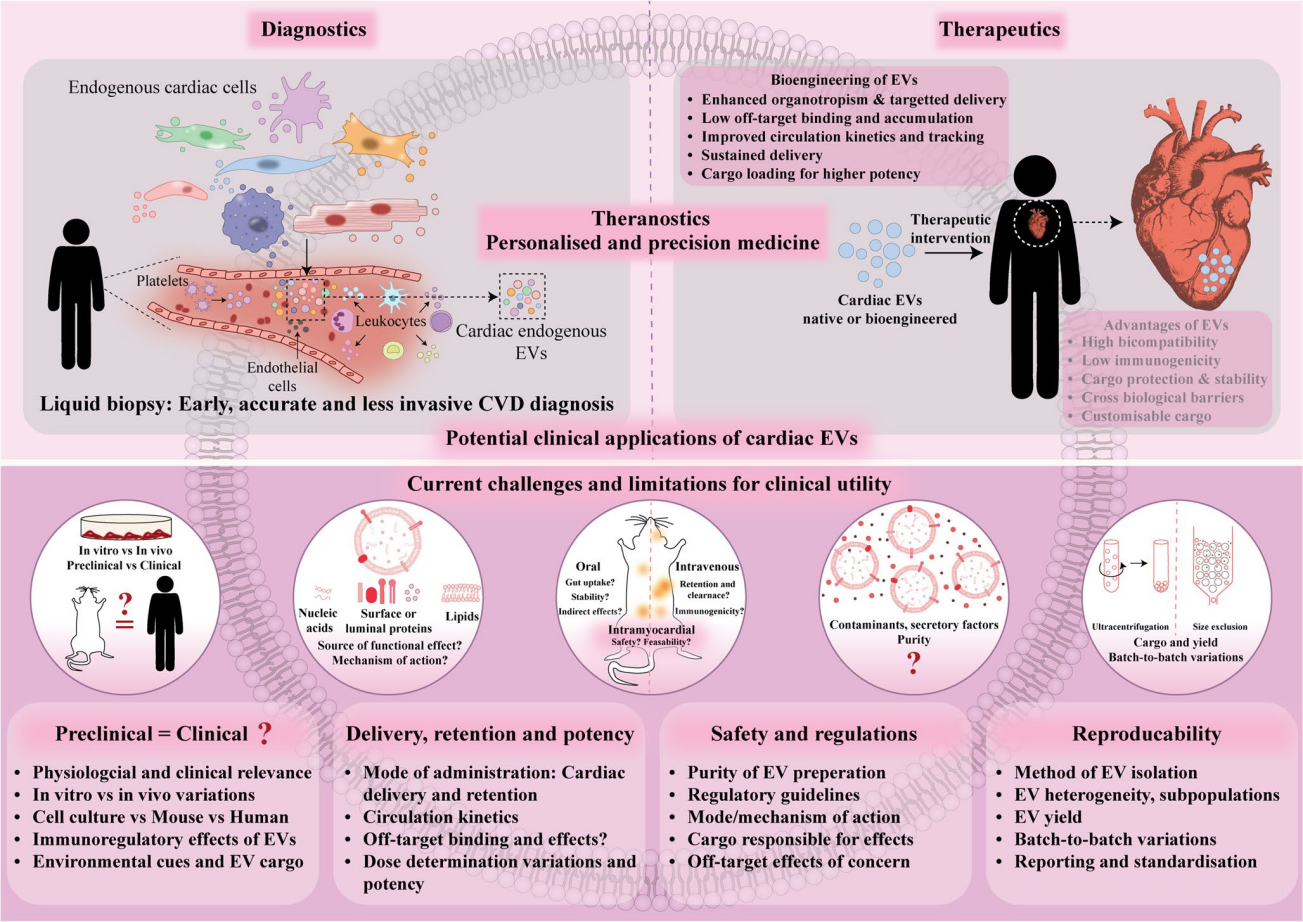
Clinical relevance of cardiac EVs. Cardiac EVs have demonstrated immense potential for utility in CVD diagnostic, prognostic and therapeutic applications. However, several challenges and limitations currently act as roadblocks and hinder their clinical translation. EV bioengineering strategies are being explored to bridge this gap from bench to bedside

From their initial discovery as mere “cellular dust” and a proposed role in cellular waste disposal, EVs have, over the past two decades, garnered substantial attention for their diverse roles in pathophysiology [167]. A growing body of literature in the past decade has highlighted their involvement in both disease progression and therapeutic modulation [7, 8, 168, 169]. This review has extensively discussed the role of EVs in cardiovascular pathophysiology. Endogenous cardiac cell-derived EVs have shown remarkable potential as mediators of cardiovascular health. In addition, EVs secreted by non-cardiac cells recruited to the heart in response to injury and inflammation also play crucial roles in disease modulation (Tables 1, 2, and 3). Current studies increasingly focus on EV bioengineering to enrich or load bioactive and therapeutic cargo for targeted delivery in various disease models [168, 170, 171]. Moreover, the enrichment of specific and distinct, native cargo under physiological and pathological conditions has positioned EVs as promising candidates for non-invasive, early disease diagnostics [172, 173] (Fig. 3).

## EVs as Biomarkers for CVD

Previous sections of this review have discussed functional implications of EVs from various cardiac endogenous cells, including CMs, CFs, ECs, and immune cells such as macrophages and neutrophils. Therefore, it is imperative to further understand cardiac endogenous EVs as signalling moieties and the mechanisms that play pivotal roles in governing CVD by regulating injury responses, immune cell activation, infiltration and recruitment, and cardiac remodelling. Studies have reported the enrichment of miRNAs in CF-EVs that are associated with CM hypertrophy, highlighting the potential utility of cardiac EVs as CVD diagnostic, prognostic and therapeutic biomarkers. For instance, miR-21-3p, miR-27a, miR-28-3p, and miR-34a have been identified in separate studies to be enriched in CF-EVs post injury and contribute to disease progression (Table 2**).** Furthermore, CFs exposed to hypoxia/reoxygenation have been shown to release EVs enriched in miR-133a, a microRNA with known cardioprotective effects [85]. Increased abundance of miR-30a in serum EVs of AMI patients has been observed, which may be released by CMs under hypoxic stimulation and is responsible for autophagy [56]. Emerging studies continue to underscore the significance of alterations in EV cargo composition in response to pathological cues, which can influence disease progression and hold prognostic potential [3, 4, 48, 169, 173]. Such findings are crucial and may inform the development of EV-based diagnostic and therefore therapeutic strategies for CVD. Tables 2 and 2, and 3 demonstrate the relevance of cardiac cell-specific EV cargo, particularly miRNAs enriched in various pathologies and cell-derived EVs, as not only potential targets for CVD therapy but also as biomarker candidates.

In this context, several studies have also shown that cardiac cell types such as CMs, ECs, and VSMCs typically secrete low levels of EVs under physiological conditions. However, upon pathological activation, their EV release is significantly enhanced [87, 90, 114]. For example, elevated levels of circulating EVs enriched with cardiac-specific miRNAs have been consistently observed following MI. A meta-analysis found that increased levels of EC-EVs in circulation were associated with higher cardiometabolic risk [96, 98].

Cardiac troponin T (cTnT) has long served as a gold-standard soluble biomarker for myocardial injury and remains integral to CVD diagnosis and patient management [174]. More recently, studies have identified cTnT within EVs isolated from both animal models of CVD and patients undergoing treatment. Using highly sensitive single-molecule quantification technologies, researchers have detected cTnT in EVs from both diseased and healthy individuals. This approach enabled molecular and biophysical profiling at the single-EV level, revealing clinically relevant differences between cTnT loaded EVs derived from patients with varying CVD etiologies and severities. While soluble cTnT levels can also be elevated in healthy individuals, EV-associated cTnT may offer improved specificity and stability, positioning it as a more robust and clinically meaningful biomarker [175]. These findings support the growing concept of EVs as “liquid biopsies,” with potential to enhance early detection, risk stratification, and personalised disease management in cardiovascular care.

Together, these findings strengthen the development and potential or cardiac cell-derived EVs as promising candidates for next-generation biomarkers in CVD. Their cell-specific origin, cargo reflective of pathological state, biophysical attributes, and presence and ease of access in biofluids such as blood position them as powerful tools for non-invasive, highly sensitive diagnostics allowing real-time monitoring and personalised CVD management.

## Extracellular Vesicles in CVD Therapy

EVs secreted by cardiac endogenous cells have been strongly positioned as promising candidates for CVD therapy, both as therapeutic agents and as targets. Studies have demonstrated that native cardiac EVs can influence disease progression by regulating key processes that impart cardioprotective effects. While preclinical studies demonstrating functional and therapeutic effects of CM- and NCM-derived EVs are summarised in Tables 2, 2 and 3, this section focuses on the broader translational potential of cardiac EVs as therapeutic agents and strategies to advance their clinical application. Enrichment of specific cargo, particularly miRNAs, plays a critical role in defining the function of EVs derived from different cardiac cell types in preclinical models of CVD (Tables 1, 2, and 3). Several of these miRNAs are known to exert cardioprotective effects. For instance, miR-133 is elevated following myocardial injury and has been associated with improved cardiac function and reduced fibrosis [85, 176–178]. However, the therapeutic application of miRNAs remains limited by challenges related to their in vivo stability and delivery. Free miRNAs are rapidly degraded by extracellular RNases and face difficulty crossing biological barriers, which compromises their efficacy. Lipid nanoparticles (LNPs) have been explored as a delivery strategy to overcome these barriers. While LNPs provide some protection and facilitate intracellular delivery, their clinical utility has been constrained by dose-limiting toxicity and preferential accumulation in the liver. These limitations have impeded their broader application in cardiovascular settings. Given these limitations of LNPs, interest has gradually shifted towards exploring EVs in CVD therapy [179, 180].

The first therapeutic utility of mesenchymal stem cell-derived EVs as CVD intervention was studied in 2010. Although several studies preceding this demonstrated the improvement in cardiac function and alleviation of CVD through stem cell transplantation, subsequent investigations demonstrated that paracrine factors, particularly EVs, were responsible for the observed therapeutic benefit. Following these developments, several studies to date have investigated the potential utility of EVs from cardiac progenitor cells as therapeutic agents in treating CVD [168, 171]. Cardiac cells under pathological stress have been shown to selectively package cardioprotective miRNAs into EVs and direct them toward target recipient cells, offering a highly regulated and physiologically relevant mode of intercellular communication (Tables 1, 2, and 3) [3, 52, 89, 168, 169, 172, 173, 181]. EVs have demonstrated the ability to cross biological barriers, including the blood-brain barrier, and protect their molecular cargo in its native state [14, 18, 182]. Moreover, EV surface molecules mediate targeted delivery, contributing to their favourable biodistribution, circulation kinetics, and cellular uptake [7, 22]. Importantly, EVs offer a biocompatible and less immunogenic alternative to their cellular counterparts [183]. Collectively, these attributes make EVs highly attractive as natural nanocarriers for therapeutic delivery in CVD. Their ability to encapsulate and deliver functional miRNAs such as miR-133 not only addresses key limitations of current delivery systems but also opens new avenues for cell-free, EV-based regenerative therapies [85]. Future research must now focus on optimising EV isolation, scalability, and functional enhancement to advance their clinical translation in cardiovascular medicine. In this regard, significant strides have been made in meeting current challenges in clinical translation of EVs using bioengineering approaches [168, 171, 173, 184]. In one such attempt to enable localised and sustained EV delivery, hydrogel patches laden with CM-EVs when implanted onto infarcted heart in rats led to cardioprotective and regenerative effects, observed as early as 24 h post-implant, ultimately leading to lower infarct size and recovery of ejection fraction [62]. The subsequent section discusses the current limitations in EV deployment in clinic for CVD therapy, emphasising more such avenues and approaches which are under investigation.

## Challenges and Limitations in Clinical Translation and EV Bioengineering for CVD Therapy

Although EVs have emerged as key regulators of cardiovascular pathophysiology and hold promise as novel therapeutic interventions for CVD, several challenges and limitations have hindered their translation into clinical application (Fig. 3). These are systematically discussed in this section.

## Preclinical Models, Standardisation and Reproducibility

Most of the current evidence supporting the therapeutic potential of EVs in CVD arises from preclinical in vitro and in vivo studies. While these findings are promising, their translational value remains limited by several critical challenges related to model relevance, EV characterization, and reproducibility [5, 14, 173, 179, 182]. One of the key challenges lies in EV heterogeneity. EVs are not a homogeneous population; they vary significantly in size, composition, and bioactivity depending on the cell of origin, the physiological state of the donor cells, and the conditions under which they are isolated [5]. Even from the same source, EVs are highly dynamic and sensitive to environmental conditions, with their cargo composition varying in response to stimuli such as hypoxia, pH shifts, nutrient availability, and cellular stress [14]. These factors can significantly influence EV function, making it difficult to compare results across studies or replicate findings. From a therapeutic perspective, identifying the EV subpopulations with desired effects is therefore paramount. MISEV2023 highlights the need for rigorous documentation of culture conditions and EV isolation parameters to enhance reproducibility and interpretability of results [5]. Additionally, many preclinical models do not accurately recapitulate human cardiac physiology or pathophysiology. Rodent models, while widely used, differ significantly from humans in cardiac anatomy, heart rate, immune response, and EV biodistribution. The dosages and routes of EV administration in animal models often exceed those feasible or safe in humans, raising concerns about clinical relevance. Another major limitation lies in the variability of EV preparation protocols [168, 171, 173, 183, 185]. Differences in isolation techniques (e.g., ultracentrifugation vs. size-exclusion chromatography), purification, storage, pre and post EV collection techniques to modulate EV bioactivity, and quantification methods leads to inconsistencies in EV yield and quality. This variation directly impacts observed bioactivity and hinders comparisons between studies. Standardized reference materials and reporting frameworks, as advocated by ISEV, are essential to address these concerns [5, 183].

## Biodistribution, Delivery and Targeting

Despite their therapeutic potential, EV biodistribution and in vivo targeting to relevant sites remains a major hurdle in their clinical translation. Depending on cell-type of EV origin, intravenous EV administration is followed by rapid clearance and accumulation in liver, spleen and lungs majorly, making their presence in circulation transient [186–188]. Organ trapping limits the amount of EVs that reach cardiac tissue, thus reducing their therapeutic efficacy as previous studies have demonstrated that increased EV efficacy is related to their retention at target site [168, 189]. Particularly, in cardiac diseases and therapy, intramyocardial delivery has been understood to have maximum cardiac retention in preclinical models. However, this might not be the clinically desirable route and method in most cases due to need for invasive catheterisation [190]. Lower cardiac accumulation is also due to lower extravasation of EVs. To achieve higher cardiac retention following intravenous dosing, higher EV dosing may be required to overcome the off-target binding. Furthermore, off-target accumulation may also have several undesirable effects [168, 191]. To overcome this challenge, EV bioengineering has been explored in several studies. Surface engineering strategies have been employed to enhance EV targeting to cardiac tissue [168, 171, 184, 192–195]. Genetic modification of parent cells to express targeting ligands—such as LAMP2B fused with myocardium-homing peptides like CSTSMLKAC—has improved EV accumulation in ischemic heart regions [196]. Alternatively, chemical conjugation approaches, including copper-free click chemistry, allow post-isolation modification of EVs with targeting peptides such as RGD (αvβ3 integrin ligand) or cardiomyocyte-specific motifs [192, 197].

## EV Tracking in Vivo

Despite growing interest in EV-mediated cardiac communication, the field still lacks a comprehensive understanding of how endogenous EVs circulate and are naturally internalised by cardiac or extra-cardiac cells in vivo. This limitation has been widely noted across preclinical studies and remains a major challenge when attempting to translate in vitro observations to physiological settings.

Typically, after intravenous administration, less than 10% of the injected (labelled) native EVs accumulate in the heart, with the biodistribution influenced by several factors, such as delivery route, EV concentration, and the source of EVs [168, 189]. The poor accumulation in the heart remains a challenge in translating EVs to clinical use for cardiovascular therapy. To complement EV bioengineering approaches for targeted cardiac delivery and enhanced organotropism, advances in tracking methods and enhanced circulation time are essential for maximizing therapeutic efficacy, particularly in cardiovascular applications. Imaging techniques such as fluorescence, luminescence, PET–MRI, and SPECT–CT are commonly used to monitor EV biodistribution. Fluorescence and luminescence are practical and widely available but offer lower sensitivity and limited quantitative capabilities. These methods are useful for tracking EVs in vitro and in vivo, but they do not provide absolute quantification or high spatial resolution. In contrast, PET–MRI and SPECT–CT offer higher sensitivity and the ability to obtain detailed anatomical images, making them more suitable for assessing EV biodistribution at higher precision [198–200].

## Safety, Immunogenicity and Regulatory Challenges

EVs are naturally occurring, physiologically intrinsic nanostructures with remarkable stability in circulation and the capacity to travel across biological barriers [5, 183]. Development of cell-based therapies has seen a recent boost, however, several concerns relating to their safety have simultaneously emerged. For instance, stem-cell base therapies present with inherent risk of neoplasm formation. Furthermore, risk of immunogenicity has impeded their long-term clinical translation [171, 201]. EVs, on the other hand, are non-replicating entities and offer advantages considering safety of administration. Their low immunogenic and toxicity inducing attributes has been extensively demonstrated in preclinical studies [5, 183, 185]. Bioengineering strategies have also been defined and are currently under exploration to address challenges related to efficient cargo loading, scalability of EV production, advancing their organotropism and potency, localised and sustained delivery, cellular uptake, enhance stability in circulation and their safety in therapeutic applications [168].

From a translational perspective, EVs are regulated as drugs and biological products by regulatory authorities, emphasising a need for transparent reporting on their manufacturing procedure, characterisation and quality control protocols [171, 173, 183]. Manufacturers currently rely on standardized pipelines for cell and protein therapy development. However, EV clinical grade therapy and translation requires unique considerations and presents challenges which are currently unmet as major regulators are yet to define detailed guidelines for EV therapeutics. Significant manufacture considerations and the challenges yet to be addressed in EV therapeutics include EV production, from selecting donor cells, determining EV potency and scalability of production to determining the EV purity, designing and implementing through characterisation of particles isolated and minimising variability with regard to storage conditions to ensure stability following isolation [171, 173, 183, 185, 202]. Although EV based therapy offers advantages in form of lower immunogenicity and toxicity, their precise mode of action remains another concern currently hindering clinical application. EVs are widely understood to have dose- and context- dependent effects. Their therapeutic benefit in preclinical models at target sites does not provide information on their long-term efficacy and safety profile systemically in other non-target locations [18, 183, 185, 203, 204]. Moreover, EV dosing remains a major area of variability and lack of consensus between groups and studies. While some dose based on particle, others define doses based on cargo such as protein [171, 185, 204]. More importantly, while preclinical studies demonstrate therapeutic effect at a certain dose or concentration, it might not accurately translate physiologically in larger organisms due to differing (lower) metabolism rate in larger species. Lastly, it has been demonstrated in a meta-analysis of preclinical investigations that there are significantly large variations between subjects with respect to EV dose and frequency of administration for achieving desired efficacy [171, 185, 204, 205]. Standardising EV dose based on their potency has been suggested in a recent study based on a specific surface cargo. This might be further beneficial in addressing batch-to-batch variations [206].

## Future Perspective

Despite current limitations that hinder their clinical deployment, continuous technological advances are steadily positioning EVs as next-generation therapeutics. The exponential growth of the EV field is evident from the surge in EV-focused publications, the hundreds of clinical trials currently underway, and the rising number of EV-centric biotech companies [168, 171, 172, 195, 207]. This review underscores the dynamic role of EVs in modulating cardiovascular pathophysiology, highlighting their functional diversity and biological relevance, particularly that of endogenous cardiac EVs. Our own studies demonstrate that myofibroblast-derived EVs can induce hypertrophic changes in human iPSC-derived cardiomyocytes, alter calcium handling and contractility through paracrine effects, and activate CFs via autocrine signalling. Proteomic analyses further reveal that these EVs carry pro-fibrotic and pro-hypertrophic cargo, providing mechanistic insight into their functional impact [208–211]. While this review concentrates on EVs secreted by resident cardiac cell populations, it is important to note that EVs from mesenchymal stem cells and other progenitor sources have shown cardioprotective and reparative potential in preclinical and translational studies. Together, these complementary lines of evidence highlight the multifaceted potential of EVs as both endogenous signalling mediators and exogenous therapeutic tools in CVD [212–217].

The accumulating body of preclinical evidence lays a strong foundation for their future clinical application. When combined with innovative bioengineering strategies—to enhance targeting, improve therapeutic potency, achieve efficient in vivo tracking, and load EVs with defined cargo—EVs hold significant promise as both therapeutic and diagnostic agents in CVD. Furthermore, establishing robust industry–academia collaborations will be critical to streamline production pipelines and harmonize regulatory frameworks for EV-based interventions. As EV research continues to evolve, these integrated efforts will be essential to bridge the gap between bench and bedside, accelerating the translation of EVs into safe, effective, scalable and personalised CVD therapeutics and for disease management.

## Data Availability

Not applicable.

## Abbreviations

BMDM: Bone marrow derived macrophage
CF: Cardiac fibroblast
CM: Cardiomyocyte
CMEC: Cardiac microvascular endothelial cells
cTnT: Cardiac troponin
CVD: Cardiovascular disease
DCM: Diabetic cardiomyopathy
EC: Endothelial cell
ECM: Extracellular matrix
EndoEVs: Endothelial cell-derived EVs
EPC: Endothelial progenitor cell
EV: Extracellular vesicle
GLUT: Glucose transporter
hiPSC-CM: Human induced pluripotent cell derived cardiomyocytes
ILV: Intraluminal vesicles
LNP: Lipid nanoparticle
MI: Myocardial infarction
miR: microRNA
MVB: Multivesicular body
NCM: Non-cardiomyocyte
PPCM: Peripartum cardiomyopathy
ox-LDL: Oxidised phospholipids
TGF-β1: Transforming growth factor-β1
TNF-α: Tumor necrosis factor-α
VSMC: Vascular smooth muscle cell
